# Determining the Thyroid Gland Volume Causing Tracheal Compression: A Semiautomated 3D CT Volumetry Study

**DOI:** 10.3390/medicina55050143

**Published:** 2019-05-16

**Authors:** Murat Binar, Mehmet Serindere, Ugur Bozlar, Serdar Karahatay, Suat Demirkapi, Umit Aydin, Mert Gokgoz, Mustafa Tasar, Mustafa Gerek

**Affiliations:** 1 Department of Otolaryngology, Head and Neck Surgery, Gulhane Medical School, Ankara 06018, Turkey; denizsea30@gmail.com (U.A.); drmcgokgoz@gmail.com (M.G.); mgerek@gmail.com (M.G.); 2 Department of Radiology, Gulhane Medical School, Ankara 06018, Turkey; drserindere@hotmail.com (M.S.); ubozlar@yahoo.com (U.B.); suatdkapi@gmail.com (S.D.); tasarmus@yahoo.com (M.T.); 3 Department of Otolaryngology, Head and Neck Surgery, Faculty of Medicine, University of Istinye, Istanbul 34010, Turkey; skarahatay@yahoo.com

**Keywords:** dyspnea, imaging, three-dimensional, organ size, thyroid gland, trachea

## Abstract

*Background and objectives:* Increased thyroid gland volume (TV) may bring about tracheal compression, which is one of the causes of respiratory distress. The aim of this study was to investigate the relationship between TV and the severity of tracheal compression independent of patients’ symptoms using semiautomated three-dimensional (3D) volumetry (S3DV) reconstructed from computed tomography (CT) scans. Cut-off TVs leading to different levels of tracheal narrowing were evaluated. *Materials and Methods:* One hundred sixty-three contrast-enhanced head and neck CT examinations were retrospectively assessed. TVs were measured by S3DV. The degree of tracheal compression was measured at the point where the greatest percent reduction in the cross-sectional area of the trachea adjacent to the thyroid gland was observed. To determine the severity of compression, the tracheal compression ratio (TCR) was defined (TCR = A1 (the narrowest cross-sectional area of trachea)/A2 (the largest cross-sectional area of trachea)). *Results*: The mean tracheal narrowing was 15% (TCR = 0.85 ± 0.15) in the study population. Patients with more than 15% tracheal compression had significantly higher TV values than those with less than 15% tracheal compression (*p* < 0.001). In addition, a significant correlation was found between TV and tracheal compression (*p* < 0.001). Moreover, the receiver operating characteristic (ROC) curve analysis revealed that the cut-off levels for TV that predict a tracheal narrowing of 10%, 20%, 30%, and 40% were 19.75 mL, 21.56 mL, 24.54 mL, and 30.29 mL, respectively (*p* < 0.05). *Conclusions:* This study objectively demonstrated that larger thyroid glands cause more severe compression on the trachea. The results may be helpful during the decision-making process for thyroidectomies to be performed due to compression symptoms.

## 1. Introduction

Respiratory distress caused by tracheal compression from the thyroid gland is an accepted indication for a thyroidectomy procedure [1,2,3,4]. Increased thyroid volume (TV) may cause respiratory symptoms due to continuous irritation of the upper airway [5], and with time, tracheal narrowing may lead to stridor and persistent hypoxia [6]. Sudden respiratory distress may also occur due to acute enlargement of the thyroid gland with intrathyroidal hemorrhage [7]. Thyroidectomy is a treatment option for patients with increased TV complicated by compression and obstruction of the trachea that is at risk of sudden onset complete airway obstruction [8,9].

When airway compromise occurs due to thyroid pathologies, decision making in regard to airway management becomes very difficult. Thyroidectomy indication for enlarged thyroid gland-causing compressive symptoms is usually based on clinical observation or a surgeon’s choice rather than on evidence-based methods [10]. The enlargement of the thyroid gland can cause different levels of airway obstruction and respiratory distress, however, precise and objective criteria to be used in the evaluation of the severity of tracheal compression have not yet been established. Although it is accepted opinion that symptoms such as dyspnea in goiter cases can resolve after thyroidectomy, the indication for thyroidectomy in cases with vague dyspnea is not clear. If one were to define these criteria, the surgical decision to perform thyroidectomy operations due to airway compression symptoms could be more evidence-based, and relief of patient symptoms after surgery could be predicted with higher accuracy.

To shed light on this issue, we investigated the relationship between thyroid gland volumes and the severity of tracheal compression using semiautomated 3D volumetry (S3DV) obtained from computed tomography (CT) scans. This possible relationship has never previously been tested through use of objective methods. In excluding patients’ symptoms from the study, we attempted to determine cut-off TVs leading to different levels of tracheal narrowing via this objective method.

## 2. Materials and Methods

### 2.1. Patients

The local ethics committee approved the study protocol (Keçiören Eğitim ve Araştırma Hastanesi Klinik Araştırma Etik Kurulu, 08.02.2017/2012-KAEK-15/1329). Records for patients who underwent neck CT and head and neck CT angiography examinations at the radiology department of a tertiary hospital between September 2014 and May 2017 were retrospectively reviewed. Subjects were excluded if they had a history of thyroid surgery or neck surgery, intratracheal lesions, tracheal stenosis due to congenital stenosis, congenital masses, tracheal anomalies, malignancies originating from neck tissues other than the thyroid gland, inflammatory diseases of the neck, and chronic pulmonary diseases that can affect the trachea. Subjects younger than 18 and older than 60 were also excluded to prevent possible bias arising from age-related tracheal alterations. Finally, 163 contrast-enhanced neck CT and head and neck CT angiography examinations were included in the study. According to the study design, all CT scans of patients were evaluated independent of respiratory symptoms. Since this was a retrospective radiological study, no informed consent was obtained from the patients.

### 2.2. Radiological Analysis

#### 2.2.1. Imaging Protocol

All of the head and neck CT angiography and contrast-enhanced neck CT examinations were obtained with a 320-row multiple detector computed tomography (MDCT) scanner (Toshiba Aquilion One, Toshiba Medical System, Otawara, Japan) using the following protocols.

The helical scanning protocol was used and images were acquired during suspended respiration for both examinations. Acquisition parameters for head and neck CT angiography were 0.5 mm slice thickness and 0.3 mm reconstruction intervals, 0.5 s gantry rotation speed, 280 mm field of view, 0.81 pitch with 80–120 kVp and automated tube current modulation. Caudocranial direction was used and scanning started from the ascending aorta to the vertex.

Nonionic iodinated contrast material at 320–400 mgI/mL was delivered at an injection rate of 5–6 mL/s, and the bolus tracking injection protocol was used. Peak density in the region of interest was selected as 180 HU at the descending aorta level. The biphasic injection for CT angiography consisted of 70–90 mL of contrast medium followed by a saline chase bolus of 30–40 mL at an injection rate of 5–6 mL/s.

Contrast-enhanced neck CT studies covering the angle of the mandible to the carina were performed after intravenous injections of 80–100 mL nonionic iodinated contrast material of 300 mgI/mL at a 4 mL/s injection rate. Scanning was initiated 70 s after the injection. The parameters used were 0.5 mm slice thickness, 0.3 mm slice interval, 280 mm field of view, 80–120 kVp and automated tube current modulation. All the radiation dose reduction methods available in the CT scanner were actively used.

Reconstructed head and neck CT angiography and contrast-enhanced neck CT images were stored in the picture archiving and communication system in our hospital.

#### 2.2.2. Image Evaluation

**Figure 1:**
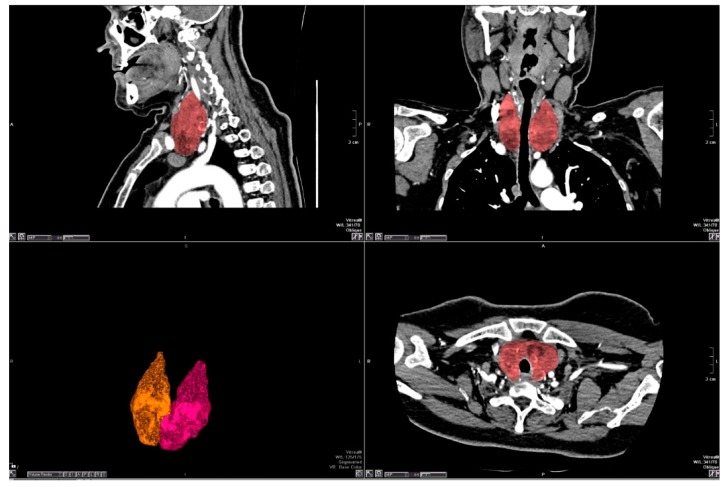
The borders of thyroid gland were drawn manually by a free region of interest (ROI) tool. The thyroid gland was extracted from other anatomic structures on three-dimensional (3D) reconstruction.

All assessments of CT examinations were made on dedicated workstations (Vitrea FX, Version 6.2, Vital Images, Minnetonka, MN, USA) in our radiology department. Segmentation of the thyroid gland was performed manually using the free region of interest (ROI) tool in all axial slices that included the thyroid isthmus and both lobes. After all the contours of the thyroid gland were marked, the software automatically deleted the anatomical structures outside the thyroid gland and only the thyroid gland became visible on the screen as a 3D volume. TV was automatically calculated by the workstation and displayed as a 3D volume-rendered CT image (Figure 1).

**Figure 2:**
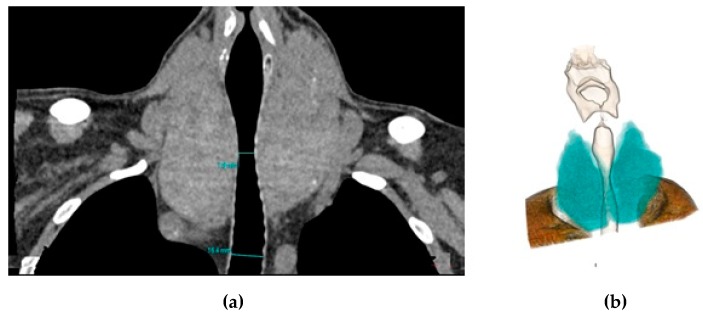
Coronal reformat (**a**) and 3D colored volume rendering (**b**) computed tomography (CT) images show the enlarged thyroid gland compressing the trachea.

The degree of tracheal compression was measured as the greatest reduction in diameter at the level of the thyroid gland observed on coronal and axial images (Figure 2).

To determine the severity of compression, the tracheal compression ratio (TCR) was defined as follows:

$$\mathrm{TCR}=\frac{A1\;\left(\mathrm{the}\;\mathrm{narrowest}\;\mathrm{cross}-\mathrm{sectional}\;\mathrm{area}\;\mathrm{of}\;\mathrm{trachea}\right)}{A2\;\left(\mathrm{the}\;\mathrm{largest}\;\mathrm{cross}-\mathrm{sectional}\;\mathrm{area}\;\mathrm{of}\;\mathrm{trachea}\right)}$$

Tracheal narrowing (%)=(1−TCR)×100

Therefore, when there is no compression on the trachea, TCR equals 1 and tracheal narrowing equals 0 (0%). Since we have calculated the area ratios to describe tracheal narrowing, the obtained values of the tracheal compression represented a more severe condition than those obtained from the calculation of diameter ratios alone.

Tracheal shift was measured from the midline and shift direction was also noted as right or left when detected.

All measurements explained above were performed blind twice for each subject by two radiologists on the same day. The average of the two measurements obtained from the two radiologists for each parameter was accepted as the true measurement of that parameter. The first radiologist had eighteen years experience and the second radiologist had six years experience in using the workstation and evaluating 3D images.

### 2.3. Statistical Analysis

Statistical analyses were performed using SPSS for Windows Ver.15.0 (SPSS Inc., Chicago, IL, USA). The Mann–Whitney *U* test was used to compare TVs between subgroups. Correlations between variables were explored using the Spearman correlation test. The receiver operating characteristic (ROC) curve analysis was performed to detect cut-off levels for TVs that indicated different levels of tracheal narrowing. In all analyses, significance was defined as *p* < 0.05.

## 3. Results

Of the total 163 patients, 69 were female and 94 were male. The mean age was 48.03 ± 14.23 years (age range, 18–60 years). The mean TV in the overall study population was 27.24 ± 31.39 mL. The mean TCR was 0.85 ± 0.14 (15% tracheal narrowing). In two patients, the minimum and maximum cross-sectional areas of trachea were the same.

In 94 male patients, the mean TV was 31.08 ± 36.87 mL and the mean TCR was 0.85 ± 0.15. In 69 female patients, the mean TV was 22.01 ± 20.96 mL and the mean TCR was 0.86 ± 0.14. The difference in the mean TV between males and females was statistically significant (*p* < 0.001) (Table 1).

**Figure 3:**
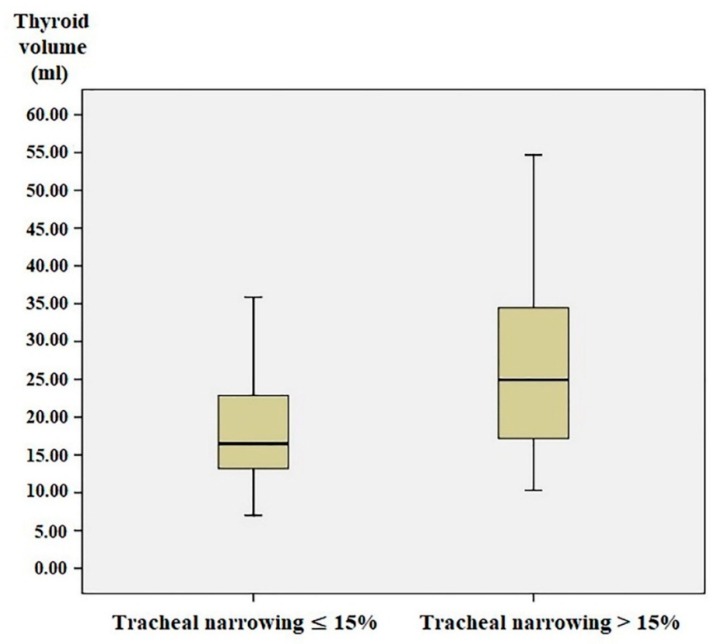
The graphic showing the thyroid volume of the patients with tracheal narrowing less than 15% and those with tracheal narrowing more than 15%. TCR, tracheal compression ratio.

According to the correlation analysis, there was a significant negative correlation between TV and TCR (*p* < 0.001; *r* = −0.324). In addition, a significant positive correlation was detected between TV and age (*p* < 0.001; *r* = 0.330). When the study population was divided into two subgroups according to tracheal narrowing of less than 15% and more than 15%, the subgroups consisted of 103 and 60 patients, respectively. The mean TV in patients with tracheal narrowing less than 15% and more than 15% were 19.77 ± 10.40 mL and 40.08 ± 47.46 mL, respectively. This difference was statistically significant (*p* < 0.001) (Table 2, Figure 3).

**Figure 4:**
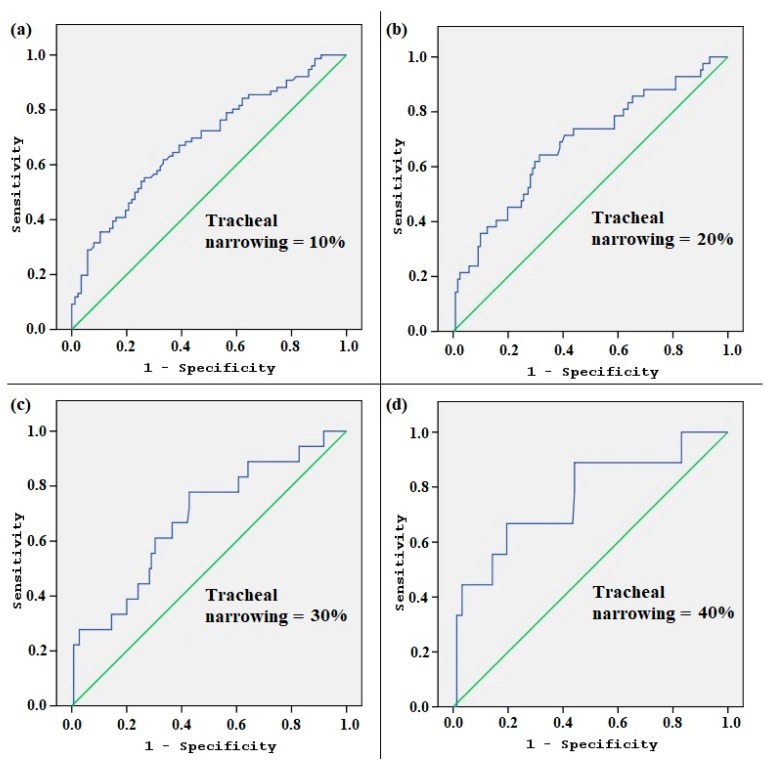
The receiver operating characteristic (ROC) curve analysis according to the different levels of tracheal narrowing. The graphics (**a**), (**b**), (**c**), and (**d**) show how the area under the curve (AUC) changes as the tracheal narrowing is adapted to 10%, 20%, 30%, and 40%, respectively.

The area under the curve (AUC) for TV according to the ROC analysis, indicating the size of the thyroid gland that causes more than 10% tracheal narrowing, was 0.684 (0.602–0.766, 95% confidence interval, *p* < 0.001). The ROC curve analysis also revealed that cut-off levels for TV that predict tracheal narrowing of 10%, 20%, 30%, and 40% were 19.75 mL, 21.56 mL, 24.54 mL, and 30.29 mL, respectively (Table 3, Figure 4).

Tracheal shift was observed in 11 patients (7%) in the range of 2–15 mm to the left or right side. There was no significant correlation between TV and the development of tracheal shift (*p* = 0.222). The mean TCR was 0.77 ± 0.09 in this tracheal shift group.

## 4. Discussion

According to the results of this study, larger thyroid glands cause more compression on the trachea. In addition, we found a significant negative correlation between TV and the cross-sectional area of the trachea. This study is the first in the literature to demonstrate a relationship between TV and tracheal compression through use of an objective method. In our study, tracheal compression due to the external effect of the thyroid gland is shown for the first time by S3DV using CT. Moreover, outcomes from the present study may provide the capability to estimate the thyroid gland volume that causes specific levels of tracheal compression.

Previous studies relevant to TV are generally based on the effect of TV on postoperative complications. Karabeyoglu et al. found that the risk of hypocalcemia increased in patients with smaller TV, whereas recurrent laryngeal nerve damage and perioperative bleeding increased in those with larger TV [11]. In the study of Karbowitz et al., patients with symptoms of respiratory insufficiency and requiring surgery due to goiter showed peak inspiratory flow rates of less than 1.5 L/s [12]. In an interesting magnetic resonance imaging (MRI) study, the smallest cross-sectional tracheal area measured by MRI was reported to be correlated with the level of respiratory function [13]. Shin et al. found a positive correlation between the size of thyroid and the presence of globus sensation and shortness of breath [14]. Our study can be complementary to those studies since deterioration in respiratory functions occurs after development of tracheal compression.

The possible causes of respiratory distress related to thyroid disease consist of four different entities: (i) intratracheal invasion of tumors, (ii) external pressure on the trachea with or without spontaneous intrathyroidal hemorrhage, (iii) compression from thyroid masses, and (iv) bilateral vocal cord paralysis due to infiltration of recurrent laryngeal nerves [15]. Of these topics, in accordance with our study design, the only issue that we looked for was the compression of the trachea from the external pressure effect of the thyroid gland. Making decisions around surgery is not easy, particularly in goiter cases with vague dyspnea. In general, surgical intervention for a large goiter may be required for several reasons: (i) prevention of complications from progressive enlargement or mediastinal extension (tracheal narrowing, superior vena cava syndrome), (ii) suspicion of malignancy, (iii) cosmetic reasons, and (iv) symptoms related to compression of the trachea or esophagus (dyspnea and/or dysphagia) [16]. Goiter may cause compression symptoms when positioned especially substernally [17], and in giant goiter cases, the thyroid is mostly retrosternally extended. However, asymptomatic benign nodular goiter may also cause acute airway obstruction, even if nodules are not large [18]. The outcomes of our study indicate that certain volumes of thyroid gland lead to different levels of tracheal compression independent of the underlying pathology.

Ultrasound (US) has been the most useful and accepted method for the estimation of thyroid volume [19,20,21]. Moreover, in emergency conditions, US can be performed quickly. However, some concerns have been reported in regard to measuring TV based on US, such as differences made among US examiners and possible inadequacy of the ellipsoid formula used for calculation of TV in the presence of nodules or irregular profiles of the gland [20,22,23]. CT and MRI are preferred by many physicians and surgeons for evaluation of thyroid gland volume when US is considered insufficient due to different sizes or properties of the thyroid gland. Although it is neither cost- nor time-effective compared to US, the CT scan has great value in the preoperative evaluation of the patient and satisfactorily shows the thyroid size, substernal and mediastinal extension of the thyroid gland and its invasion into surrounding tissues and organs [4,24]. Previous studies have shown that, except for cases with substernal goiter, TV measured by US did not differ significantly from that measured by CT [25,26]. In the study of Lee et al., when the true thyroid volume was considered to be the volume of the excised thyroid specimen measured by the water displacement method, their results showed no significant difference between thyroid specimen volumes and the 2D US volumes or between thyroid specimen volumes and 3D CT volumes, whereas there was a significant difference between the thyroid specimen volumes and the 2D CT volumes [27]. Choi et al. observed a good correlation between the weight of the postoperative thyroid specimen and the spiral CT volumetry results of the thyroid gland in patients with Graves’ disease. They also reported that minimally invasive thyroid surgery with less blood loss can be possible when the estimated TV with CT volumetry is less than 100 mL [28]. Beyond these, CT is favored to US in its assessment of tracheal compression, especially in cases with a retrosternal extending thyroid. However, the problem with CT is that it involves radiation and is costlier than US in thyroid volumetric evaluation. Therefore, cut-off values of TV calculated in this study can be used in the future as a reference for volume evaluation and can be adapted to US in most cases without substernal goiter.

In the overall study population, left- or right-sided tracheal shift was observed in 11 patients, determined according to the deviation distance from the midline. Since this is a retrospective radiological study, the correlation of patients’ symptoms with tracheal shift was unable to be assessed. In the study of Shin et al., tracheal compression but not deviation was found to be related to shortness of breath [14]. Our results have also showed that there is no significant correlation between TV and development of tracheal shift. On the other hand, the mean tracheal narrowing was 23% (TCR = 0.77) in this tracheal shift group. Compared to average of all patients (TCR = 0.85), we see that tracheal deviation due to unilateral huge goiter affects TCR more. In patients with long-lasting tracheal compression due to a large goiter, tracheomalacia can develop. These patients usually experience stridor, however, it is not common in goiter cases that tracheomalacia needs to be repaired by means of tracheal resection [16,29].

One important issue is use of contrast while performing CT. In some cases, non-contrast neck CT scans may be sufficient to evaluate a thyroid gland that shows benign properties and does not extend substernally. If contrast is given to make a detailed evaluation in a patient scheduled for surgery, since contrast carries high iodine content, surgical intervention is recommended to be performed within one month to avoid possible hyperthyroidism. Moreover, if the patient has thyroid nodules suspicious for follicular cell thyroid cancer, intravenous contrast should not be used as it will lead to a delay in radioactive iodine therapy due to its high iodine content [16,30,31]. In our study, contrast-enhanced CT scans were examined. The S3DV can be adapted to both contrast and non-contrast CT scans, however, the presence of contrast helps the radiologist to distinguish surrounding tissues from the thyroid gland more accurately.

We need to mention the limitations of this study. First, the nature of the study is retrospective, since it is somewhat difficult to design a prospective CT study due to ethical issues. Second, this retrospective study was designed as a radiological study independent of histopathological evaluation of the thyroid gland, as different tissue patterns of thyroid pathologies may cause different levels of compression patterns on the trachea. It is important to know whether the goiter is diffuse or nodular, and the elasticity of the nodules may also be different. Even the presence of chronic thyroiditis can change the hardness of the gland. None of the patients included in this study had diffuse or infiltrative diseases such as anaplastic carcinoma, thyroid lymphoma or diffuse epithelial thyroid cancer. Third, the absence of inter/intra-rater reliability work was another limitation of our study. Fourth, as we mention above, the main purpose of this study was to evaluate the effect of TV on tracheal compression independent of patients’ symptoms. The symptoms may occur due to many factors such as the duration of the compression, the condition of the lower airways, the lung parenchyma, and the expanding ability of the thorax. In addition, tracheal cartilage and laryngeal cartilages show ossifications as age progresses that lead to more resistance from external compression. All these variables are outside the scope of this study. We investigated this relationship in a group of patients whose CT measurements were retrospectively included to detect the cut-off value for TV that causes tracheal compression. The outcomes of this study may be helpful in estimating tracheal narrowing by measuring TV even with the use of US, which is simpler to work with compared to CT. Forthcoming prospective studies may also investigate the degree of tracheal compression combined with assessment of respiratory function tests to reveal how patients become symptomatic as the thyroid gland enlarges.

## 5. Conclusions

This study objectively demonstrates that larger thyroid glands cause more severe compression on the trachea. This relationship can predict cut-off levels of thyroid volumes that cause different levels of tracheal narrowing, which are values that can be used as a future reference for volume evaluation and can be adapted to easier imaging methods like US. As a result, the outcomes of this study are considered to be helpful in the decision-making process for thyroidectomies to be performed due to compression symptoms.

## Figures and Tables

**Table 1:** The general data according to sex.

	Male (*n* = 94)	Female (*n* = 69)	*p* Value
The mean age	46.15 ± 14.25	50.57 ± 13.90	0.070
The minimum TV	7.00	7.31	N/A
The maximum TV	249.42	138.27	N/A
The mean TV	31.08 ± 36.87	22.01 ± 20.96	<0.001
The minimum TCR	0.06	0.06	N/A
The maximum TCR	1.00	1.00	N/A
The mean TCR	0.85 ± 0.15	0.86 ± 0.14	0.954

TV, thyroid volume; TCR, tracheal compression ratio; N/A, not applicable.

**Table 2:** Comparison of the mean TV between patients with tracheal narrowing more than 15% and those with less than 15%.

	(1 − TCR) × 100 ≤ 15%	(1 − TCR) × 100 > 15%	*p*
The mean TV (mL)	19.77 ± 10.40	40.08 ± 47.46	<0.001

**Table 3:** Cut-off values of TV relative to different levels of tracheal narrowing.

(1 − TCR) × 100 (Tracheal Narrowing)	Cut-Off (TV)	AUC	Lower Bound	Upper Bound	*p*
>10% (*n* = 76)	19.75 mL	0.684	0.602	0.766	<0.001
>20% (*n* = 42)	21.56 mL	0.683	0.586	0.780	<0.001
>30% (*n* = 18)	24.54 mL	0.682	0.548	0.816	0.012
>40% (*n* = 9)	30.29 mL	0.764	0.587	0.942	0.008

AUC, the area under the curve.
